# A Rare Clinical Presentation of Giant Bilateral Labial Fibroepithelial Stromal Polyps in Patient with Psoriasis Disease

**DOI:** 10.1155/2016/7942365

**Published:** 2016-01-26

**Authors:** Ayse Filiz Avsar, Elcin Islek, Melahat Yildirim, Hilal Ahsen

**Affiliations:** ^1^Department of Obstetrics and Gynecology, Yildirim Beyazit University, Bilkent, 06800 Ankara, Turkey; ^2^Department of Obstetrics and Gynecology, Ankara Ataturk Training and Research Hospital, Bilkent, 06800 Ankara, Turkey; ^3^Department of Pathology, Ankara Ataturk Training and Research Hospital, Bilkent, 06800 Ankara, Turkey

## Abstract

Fibroepithelial polyps (FEPs) are rarely seen lesions of the lower female genital tract with polypoid proliferations of stroma. These tumors usually present in the vulvovaginal region of the reproductive aged women. In this presentation, we report a case of a psoriatic woman who developed unusual multiple polypoid lesions approximately 15 cm in size arising from both left and right labia minora and unique connection of FEPs with psoriasis disease.

## 1. Introduction

Fibroepithelial polyps (FEPs) are rarely seen lesions of the lower female genital tract with polypoid proliferations of stroma [1, 2]. These tumors usually present in the vulvovaginal region of the reproductive aged women and tend to be polypoid or pedunculated in appearance and mostly found as solitary masses in the lower female genital tract. When associated with pregnancy, they commonly present multiple lesions with a tendency of cellular pleomorphism [3]. FEPs are found to be related to certain diseases such as Crohn's disease and congenital lymphedema [4, 5].

In this presentation, we report a case of a psoriatic woman who developed unusual multiple polypoid lesions approximately 15 cm in size arising from both left and right labia minora and unique connection of FEPs with psoriasis disease. Written informed consent was obtained from the patient according to the tenets of the Declaration of Helsinki. After surgical resection, histopathological investigation confirmed the diagnosis of FEP.

## 2. Case Presentation

**Figure 1:**
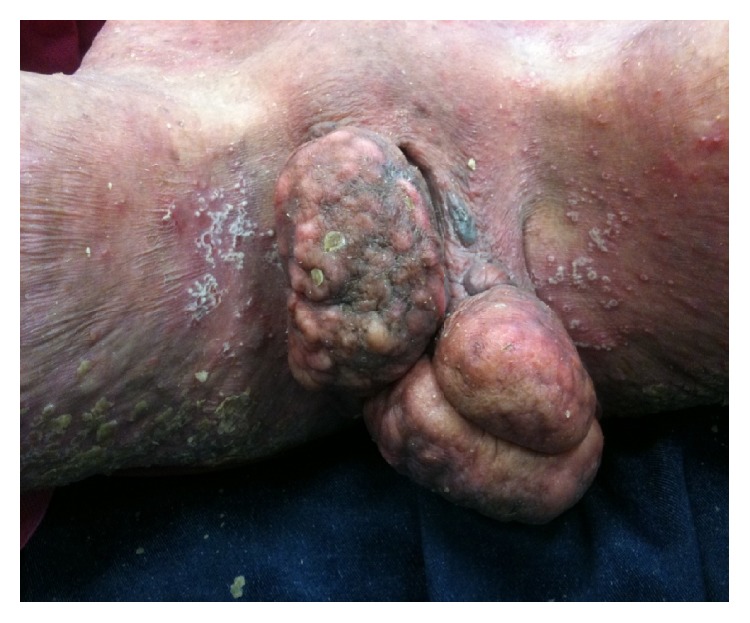
Giant bilateral labial fibroepithelial stromal polyps in patient with psoriasis disease.

**Figure 2:**
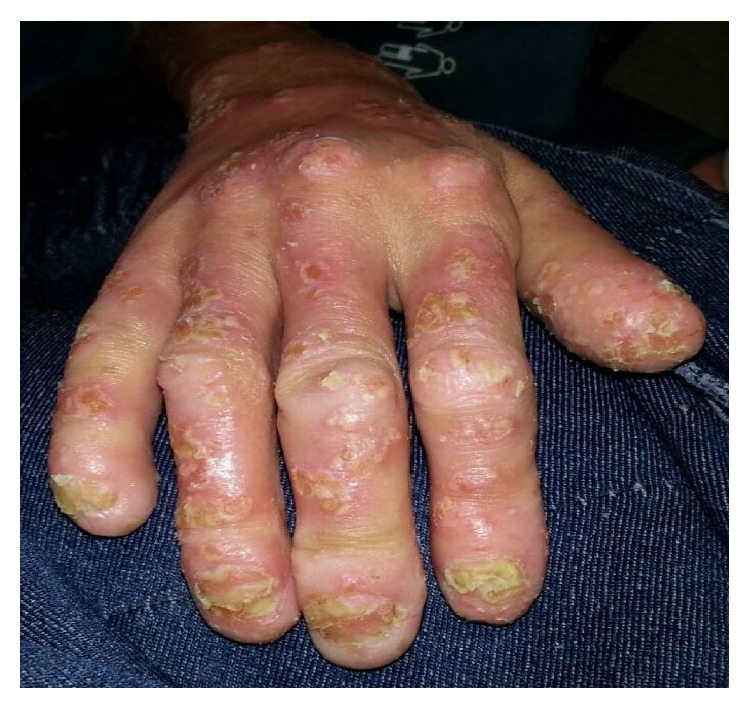
Nail dystrophies and finger joint deformities associated with psoriasis.

**Figure 3:**
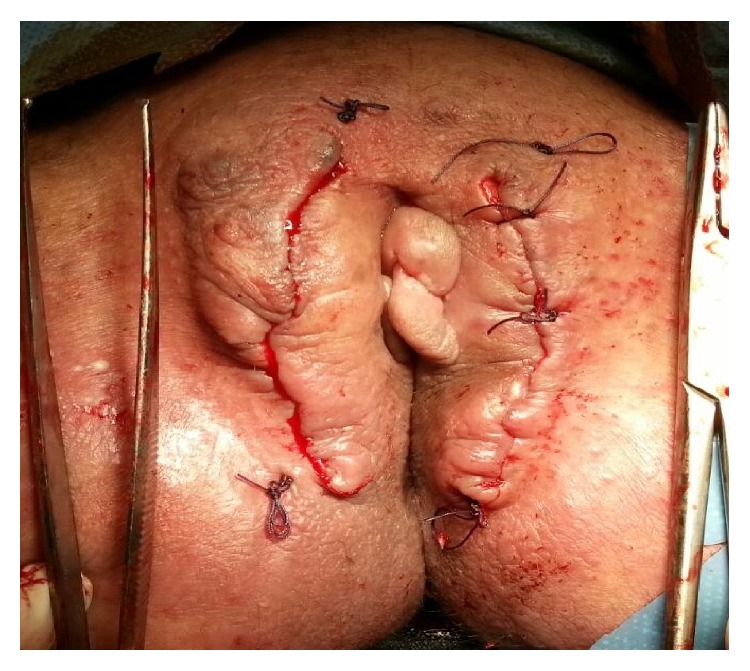
Labial masses were removed surgically.

**Figure 4:**
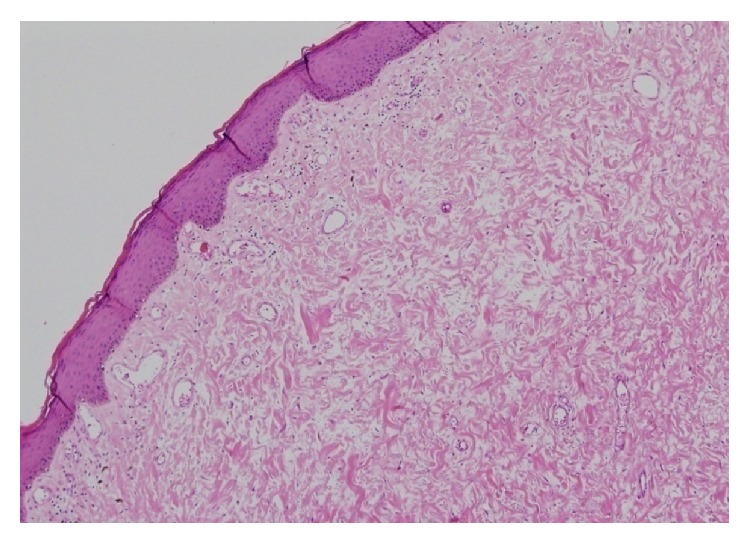
Fibroepithelial stromal polyp with a hypercellular and focal myxoid stroma and vascular structures (hematoxylin and eosin stain, magnification ×100).

A 28-year-old nulligravid woman with a history of psoriasis disease admitted to our gynecologic clinics complaining of bilateral painless vulvar masses which were first noticed five years ago. Until then, the patient did not seek any treatment for these slow-growing masses due to some personal reasons. But, over the course of 12 months, the labial masses have grown gradually and she decided to seek treatment because of a sudden increase in the size of labial masses. Her medical history was not remarkable for any other diseases rather than psoriasis which she has had since she was born. Gynecological examination revealed two separate pedunculated, nontender polypoid lesions approximately 15 × 10 cm and 13 × 6 cm in sizes arising from both left and right labia minora (Figure 1) accompanying generalized erythematous papules, plaques, and dry scales with typical clinical presentation of psoriasis disease (Figure 1). In addition, nail dystrophies and finger joint deformities associated with psoriasis were observed in the patient (Figure 2). She denied experiencing any trauma, drug usage, or infection. Labial masses were removed surgically (Figure 3) and sent for histopathological investigation. Histological examination of polypoid lesions showed spindle-shaped cells, mesenchymal stellate cells, and frequently multinucleated cells in a focal myxoid stroma (Figure 4) which were typical for FEPs.

Some lesions were found as moderately hypercellular. The surface of the polypoid lesion showed the fern-like papillary appearance covered by intact epidermis. In addition, chronic inflammation was detected in the pathological specimen. Immunohistochemical staining was negative for S100, CD-34, and cytokeratins, and the involved areas seemed to be immunoreactive for desmin.

## 3. Discussion

Psoriasis is a chronic inflammatory skin disease involving hyperproliferation of the keratinocytes in the epidermis. The typical clinical presentation of the disease is itchy, erythematous lesions covered with silvery scales on patients skin as a result of an increased epidermal cell turnover rate [6]. 29–40% of patients with psoriasis diseases have genital skin lesions accompanied by generalized erythematous papules [7, 8]. Psoriasis is responsible for 2% of chronic symptomatic vulvar diseases [9, 10] as typically exhibiting symmetrical erythematous, irregular shaped papules in the vulvovaginal region. It is not uncommon to see fissures, exudation, and maceration associated with itching and burning sensation in these lesions [11]. Since scar formation is extremely rare in genital psoriasis, there is only one case in literature with respect to vulvar scar formation in the psoriatic patient [12].

FEPs are extremely rare, benign, and locally infiltrative tumors with pedunculated polypoid appearances originating from pelvic soft tissues [13]. They mostly grow being less than 5 cm in diameter [14], but they can rarely reach extremely large size, up to 15–20 cm. A few reports regarding the giant FEPs larger than 10 cm have been described in literature [5, 15, 16]. Of all these reports, there was only one case except our presentation reporting the labial FEP in the psoriatic patient [15]. However, current case report apparently differs from the first one in describing the extremely rare coexistence of multiple large FEPs arising from both left and right labia minora in the patient.

FEPs are prone to circumscribed lesions with central fibrovascular cores in them and they mostly occur in the superficial subepithelial stroma containing stellate and multinucleated stromal cells [13]. Immunohistochemical staining was positive for desmin, vimentin, estrogen, and progesterone receptors, and less commonly alpha smooth muscle actin [17]. The staining was negative for S100 and cytokeratins [17] as observed in our case report. The presence of estrogen and progesterone receptors in the lesion refers to the hormonal influence on the development of polyps. Furthermore, the findings that the regression of lesions in the postpartum period, existence of polyps in postmenopausal women on hormone replacement therapy, and being rare in premenarchal and postmenopausal women confirm the hormonal influences on the development of FEPs [3].

Although the pathogenesis of FEPs is not completely enlightened, the reactive hyperplastic process in subepithelial mesenchymal tissues in the genital region is considered to play an important role in the pathogenesis of FEP [18]. In the case report of Dane et al., they have proclaimed that chronic inflammatory processes as in psoriasis diseases may modulate or trigger the development of FEPs under the influence of reparative processes similar to neoplasms [15]. In the case report of Navada et al., they detected the inflammation in their pathological specimen; however, they interpreted these findings as inflammation that occurred as a result of a possible secondary infection following trauma [16]. In the current case, histopathological examination revealed signs of inflammation in FEPs too.

FEPs typically present as single lesions in general, but they have the propensity of being multiple during pregnancies [13]. In current case report, we present the multiple FEPs in nonpregnant woman in whom FEP should appear as a solitary lesion. The woman subject to this report admits that she have not adhered to treatment of psoriasis so far and she has never been disease-free in her life.

Based on the knowledge that the patient's denial of experiencing conditions which might cause vulvar FEPs such as the history of being pregnant, using tamoxifen or hormonal therapy, and being exposed to repeated or long-standing trauma and the patient's approval for the period when FEP occurred and grown rapidly which coincided with the time in which psoriasis worsened and became uncontrolled, we regard that this young woman developed multiple vulvar FEPs caused by an uncontrolled psoriasis disease.

In conclusion, this case presentation is the second reported case with respect to the psoriatic patient with FEP, but it differs from the first one in presenting the rarely seen bilateral multiple FEPs in the lower female genital tract. It is hoped that this work will make significant contribution to literature on FEP development in psoriatic patients.
